# Efficient Ensemble Learning with Curriculum-Based Masked Autoencoders for Retinal OCT Classification

**DOI:** 10.3390/diagnostics16020179

**Published:** 2026-01-06

**Authors:** Taeyoung Yoon, Daesung Kang

**Affiliations:** School of Bio-Health Convergence, College of Natural Sciences, Sungshin Women’s University, Seoul 01133, Republic of Korea; yty5957@naver.com

**Keywords:** curriculum learning, ensemble learning, masked autoencoders, model soups, optical coherence tomography, self-supervised learning, snapshot ensemble

## Abstract

**Background/Objectives**: Retinal optical coherence tomography (OCT) is essential for diagnosing ocular diseases, yet developing high-performing multiclass classifiers remains challenging due to limited labeled data and the computational cost of self-supervised pretraining. This study aims to address these limitations by introducing a curriculum-based self-supervised framework to improve representation learning and reduce computational burden for OCT classification. **Methods**: Two ensemble strategies were developed using progressive masked autoencoder (MAE) pretraining. We refer to this curriculum-based MAE framework as CurriMAE (curriculum-based masked autoencoder). CurriMAE-Soup merges multiple curriculum-aware pretrained checkpoints using weight averaging, producing a single model for fine-tuning and inference. CurriMAE-Greedy selects top-performing fine-tuned models from different pretraining stages and ensembles their predictions. Both approaches rely on one curriculum-guided MAE pretraining run, avoiding repeated training with fixed masking ratios. Experiments were conducted on two publicly available retinal OCT datasets, the Kermany dataset for self-supervised pretraining and the OCTDL dataset for downstream evaluation. The OCTDL dataset comprises seven clinically relevant retinal classes, including normal retina, age-related macular degeneration (AMD), diabetic macular edema (DME), epiretinal membrane (ERM), retinal vein occlusion (RVO), retinal artery occlusion (RAO), and vitreomacular interface disease (VID) and the proposed methods were compared against standard MAE variants and supervised baselines including ResNet-34 and ViT-S. **Results**: Both CurriMAE methods outperformed standard MAE models and supervised baselines. CurriMAE-Greedy achieved the highest performance with an area under the receiver operating characteristic curve (AUC) of 0.995 and accuracy of 93.32%, while CurriMAE-Soup provided competitive accuracy with substantially lower inference complexity. Compared with MAE models trained at fixed masking ratios, the proposed methods improved accuracy while requiring fewer pretraining runs and reduced model storage for inference. **Conclusions**: The proposed curriculum-based self-supervised ensemble framework offers an effective and resource-efficient solution for multiclass retinal OCT classification. By integrating progressive masking with snapshot-based model fusion, CurriMAE methods provide high performance with reduced computational cost, supporting their potential for real-world ophthalmic imaging applications where labeled data and computational resources are limited.

## 1. Introduction

Deep learning has made significant advances in medical image analysis, largely due to its ability to automatically extract complex hierarchical features and outperform traditional methods across a wide range of diagnostic tasks [1,2,3]. However, the effectiveness of supervised deep learning models heavily depends on the availability of large-scale annotated datasets, which are often difficult or expensive to obtain in clinical practice [3,4]. This challenge has motivated a growing interest in self-supervised learning (SSL), which enables models to learn transferable visual representations from unlabeled data by designing pretext tasks [5,6].

Among the diverse SSL paradigms, masked image modeling (MIM) has recently emerged as a powerful approach, with masked autoencoders (MAE) standing out as a leading framework [7,8,9]. MAE reconstructs masked regions of input images from the remaining unmasked patches, encouraging the MAE encoder to capture both global context and semantic information [9]. Typically, an MAE consists of a vision transformer (ViT) encoder and a lightweight decoder. It has demonstrated strong performance across various downstream tasks including classification, segmentation, and detection [9,10,11].

To further improve the effectiveness of MIM training, curriculum learning-based masked autoencoders (CurriMAE) was recently introduced [12]. CurriMAE progressively increases the masking ratio during pretraining, allowing the model to start with easier reconstruction tasks and gradually adapt to more challenging ones. This curriculum-based strategy has been shown to enhance representation learning and outperform MAE models trained with a fixed masking ratio in multi-labeled classification of pediatric thoracic disease from chest X-ray images [12].

While CurriMAE improves representation quality, there remains potential to further boost downstream task performance through ensemble learning. One effective approach is snapshot ensembling, where intermediate model checkpoints (“snapshots”) are saved at predefined intervals during pretraining, each corresponding to different masking ratios. In prior work, these snapshots were independently fine-tuned and their outputs averaged at inference, leveraging the diversity of learned representations to enhance robustness and generalization [13]. However, this approach suffers from substantial computational overhead, as it requires multiple fine-tuning processes, increased storage for all models, and multiple forward passes during inference.

To address these issues, we explore two strategies that leverage pretrained snapshots with improved efficiency: CurriMAE-Soup and CurriMAE-Greedy, which differ in their trade-offs between training cost and performance. CurriMAE-Soup merges the weights of multiple pretrained snapshots into a single model using the model soup technique, enabling fine-tuning and inference with only one model while retaining the predictive advantages typically associated with ensemble methods. CurriMAE-Greedy selects the top-performing fine-tuning of selected snapshots based on validation performance and ensembles only a small subset (two or three models, denoted as CurriMAE-GE2 and CurriMAE-GE3, respectively). Although CurriMAE-Greedy still requires separate fine-tuning of selected snapshots, it reduces storage and inference costs compared to full snapshot ensembles. We evaluate the effectiveness of these methods on optical coherence tomography (OCT) classification task, demonstrating that they achieve competitive or superior performance across all major evaluation metrics, including AUC, AUPRC, accuracy, sensitivity, precision, and F1-score compared to standard MAE and CurriMAE, while improving inference efficiency.

The main contributions of this work are as follows:

• We introduce CurriMAE-Soup, which combines multiple pretrained snapshots into a single model using the model soup technique, eliminating the need for multiple fine-tuning processes and enabling efficient inference.
• We propose CurriMAE-Greedy, which ensembles only the top-performing fine-tuned snapshots (two or three models), reducing storage and inference costs compared to full snapshot ensembles while maintaining high performance.
• We conduct experiments on OCT classification, demonstrating that both CurriMAE-Soup and CurriMAE-Greedy achieve competitive or superior performance compared to other methods.
• We analyze the trade-offs between performance and computational efficiency across different ensemble strategies, providing practical insights for deploying SSL models in clinical applications.

## 2. Related Works

### 2.1. OCT Disease Classification with Deep Learning

Deep learning has emerged as a transformative approach for analyzing OCT images, enabling automated detection and classification of various retinal diseases [14,15,16]. Leveraging large-scale labeled datasets and deep learning networks, these methods have achieved expert-level performance in diagnostic tasks [2,14].

Kermany et al. developed a deep learning framework for automated diagnosis of common treatable ophthalmic diseases using a large-scale OCT dataset. The model, based on the Inception-V3 architecture, was trained to classify images into categories such as normal retina, diabetic macular edema (DME), choroidal neovascularization (CNV), and drusen. Using 108,312 OCT images, the system achieved diagnostic performance comparable to that of retina specialists [14]. Ardelean et al. proposed a comprehensive evaluation of state-of-the-art object detection models—particularly recent YOLO variants—for identifying retinal pathologies in OCT scans. By benchmarking YOLOv8–YOLOv12, YOLO-World, YOLOE, and RT-DETR on two OCT datasets (AROI and OCT5k), the study demonstrated that YOLOv12 achieves the best balance between accuracy and computational efficiency, while YOLOE consistently delivers the highest detection performance across most pathology classes. Their results emphasize the growing potential of modern object detectors for automated medical OCT analysis [15]. Miladinović et al. evaluated several pre-trained deep learning architectures, including ResNet and Inception-V3, for classifying retinal pathologies versus healthy cases using OCT images under conditions of data scarcity and label noise. They found that all models achieved classification accuracy of at least 90% when trained on 345 or more images, while mislabeled data significantly degraded performance unless offset by a proportionally larger training set [16].

### 2.2. Masked Image Modeling for OCT

MIM has emerged as a powerful SSL paradigm in which parts of the input image are masked and the model learns to reconstruct the missing content. Inspired by masked language modeling in natural language processing (e.g., BERT), MIM encourages models to learn semantically meaningful representations from unlabeled data by predicting missing image regions [7,8,9,10,17,18,19,20].

Pissas et al. introduced a large-scale MAE pretraining framework using over 700 K OCT images from 41 K patients, covering diverse retinal diseases. Their approach demonstrated that MIM-pretrained representations significantly improved performance on six downstream tasks, including disease classification, biomarker detection, and fluid/layer segmentation. They also proposed a multimodal extension combining OCT and infrared fundus images, showing additional benefits in multimodal downstream tasks [18]. Wang et al. introduced a dynamic masking strategy and a parallel CNN-Transformer encoder for MAE pretraining on gynecological OCT images. The proposed method improved classification accuracy, robustness, and interpretability in low-label settings, while reducing training cost in downstream tasks [19]. Yoon and Kang proposed Self-Distilled Masked Autoencoders (SD-MAE) to enhance feature learning in self-supervised settings by introducing pseudo-label–based self-distillation into the MAE encoder. Applied to multiclass retinal OCT classification, SD-MAE achieved AUC 0.995, outperforming ResNet-34, ViT-S, and standard MAE baselines while requiring no additional annotated data [20].

### 2.3. Ensemble Learning and Efficient Model Aggregation Strategies

Ensemble learning is a long-established approach for improving predictive accuracy and robustness by combining multiple models. Traditional strategies such as bagging, boosting, and voting aggregate outputs from independently trained models to reduce variance and improve generalization [21,22,23]. In deep learning, ensemble methods often exploit model diversity by training with different random seeds, varying data partitions, or sampling checkpoints from different points in the optimization trajectory. While effective, these approaches incur substantial computational and storage costs, as multiple models must be maintained and evaluated during inference [13,24,25,26].

Snapshot ensembling, introduced by Huang et al., addresses some of these costs by saving model weights at different local minima along a cyclic cosine annealing learning rate schedule within a single training run [13]. This produces multiple diverse “snapshots” without training from scratch each time, which can then be ensembled to boost accuracy. Building on this, Geetha et al. proposed DEEP-GD, a glaucoma detection framework that integrates snapshot ensembles with EfficientNet-based CNNs. By saving model weights from different local minima within a single training run and ensembling them, they improved diagnostic accuracy (average 99.35%) and robustness compared to a single model. In addition, for positive glaucoma detections, they performed disease staging using a V-Net segmentation model. This work presents the effectiveness of snapshot ensembles for fundus-based disease classification [27]. Yoon and Kang proposed a self-supervised snapshot ensemble framework (Snap-MAE) for medical image classification, leveraging cyclic cosine learning rate schedules to capture diverse representations during a single pretraining run. They demonstrated superior performance and robustness on pediatric thoracic and cardiovascular disease datasets by ensembling pretrained snapshots obtained from a self-supervised ViT-based MAE. Their method effectively bridges the gap between SSL and ensemble strategies in clinical imaging tasks [28]. Snapshot ensembles have been successfully applied to both supervised and self-supervised tasks, including medical image analysis, but still require multiple fine-tuned models and multiple forward passes at inference.

To further reduce inference cost, weight-space aggregation methods such as model soups have been proposed [29,30]. Wortsman et al. demonstrated that averaging the weights of multiple fine-tuned models initialized from the same pretrained checkpoint can yield performance comparable to prediction-level ensembles while maintaining the efficiency of a single model at inference [30]. This makes model soups particularly appealing for deployment scenarios where storage and computational resources are limited. Extending this concept to the medical domain, Maron et al. applied model soup techniques to dermoscopic skin cancer classification, showing that weight averaging across multiple fine-tuned classifiers improved performance and robustness over individual models. Their results emphasize the potential of model soups to enhance generalization in high-stakes medical image analysis while avoiding the high computational and storage demands of traditional ensembles [31].

## 3. Materials and Methods

### 3.1. OCT Datasets

To facilitate self-supervised representation learning and subsequent supervised classification of retinal diseases, we utilized two publicly available OCT datasets: the Kermany OCT dataset for pretraining and the OCTDL dataset for downstream fine-tuning [14,32]. These datasets differ significantly in size, labeling granularity, and diagnostic focus, making them complementary for building and evaluating robust OCT classification models. Table 1 summarizes the class-wise distribution of training, validation, and test samples in the OCTDL dataset. The variation in sample size across disease categories introduces class imbalance, which we address through weighted and macro-averaged evaluation metrics.

The Kermany OCT dataset, originally released by Kermany et al., contains 108,309 B-scan images categorized into four broad diagnostic classes: normal, DME, CNV, and drusen [14]. Although the dataset includes class labels, they were not used during pretraining. Instead, we treated the dataset as unlabeled and used it exclusively for SSL under a MAE framework. Its large volume and wide variability in retinal anatomy made it ideal for learning general-purpose visual representations.

**Figure 1:**
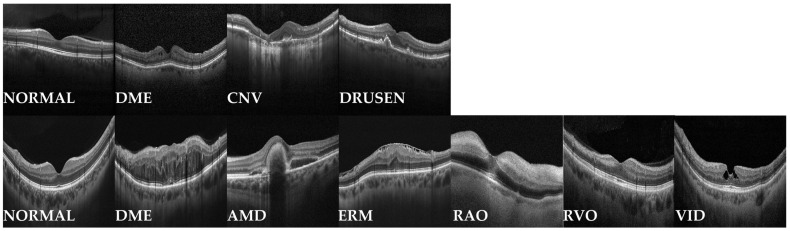
Representative OCT images from the Kermany and OCTDL dataset. The first row shows samples from the Kermany dataset, which was employed for self-supervised pretraining. The second row presents samples from the OCTDL dataset, used for supervised fine-tuning. Labels are indicated below each image.

For supervised fine-tuning and evaluation, we employed the OCTDL dataset, which consists of 2064 high-resolution OCT images annotated with seven distinct retinal conditions: normal (NOR), DME, AMD, epiretinal membrane (ERM), retinal artery occlusion (RAO), retinal vein occlusion (RVO), and vitreomacular interface disease (VID) [32]. The data was divided into 1660 training samples and 404 test samples, and we allocated 20% of the training data for validation. This resulted in class-wise splits for training, validation, and testing, as detailed in Table 1. Compared to the Kermany dataset, OCTDL provides more fine-grained pathology labels, allowing evaluation of multiclass classification performance in a clinically realistic setting. Sample images from both the Kermany OCT and OCTDL datasets are presented in Figure 1.

### 3.2. Pretraining: Progressive Masking with MAE via Curriculum Learning

**Figure 2:**
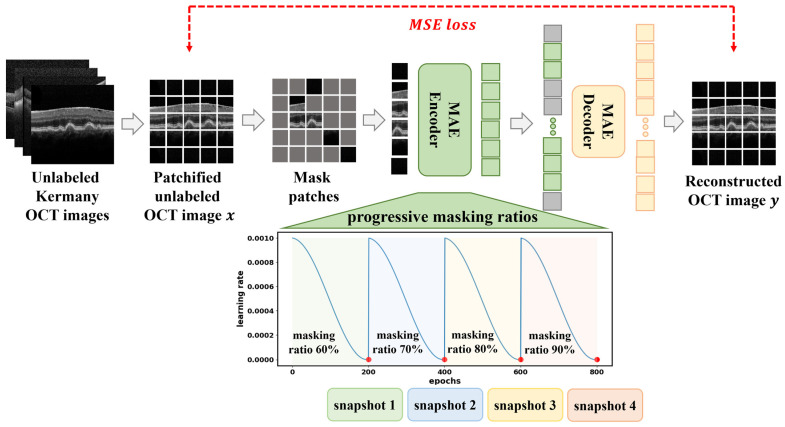
Overview of the proposed curriculum-based MAE pretraining framework using the Kermany OCT dataset. OCT images are tokenized into non-overlapping patches, with a progressive increase in masking ratio from 60% to 90% across four training stages. The cyclic cosine learning rate schedule encourages diverse model snapshots, which are later used for ensembling. The colored dots indicate the snapshot saving points at the end of each curriculum stage, with different colors used to distinguish successive stages.

We extend the standard MAE framework by incorporating a curriculum-based progressive masking strategy to guide the model from easier to harder reconstruction tasks [9,12]. This approach, illustrated in Figure 2, is designed to gradually increase the difficulty of the learning objective, thereby improving the quality and robustness of learned visual representations.

In the original MAE setup, an input image of size H×W is divided into non-overlapping patches of size p×p (typically 16×16), resulting in a total of $N=\left(H\times W\right)/p^{2}$ tokens. A subset $M\subset \left\{1,\ldots ,N\right\}$ of these tokens is randomly masked, while the remaining visible tokens are passed to a ViT-S encoder. A lightweight decoder reconstructs the masked tokens using information from the encoded visible tokens. The objective is to minimize the reconstruction error over the masked tokens only, using the mean squared error (MSE) loss:

(1) $$L_{MSE}=\frac{1}{\left|M\right|}\sum_{i\in M}{\left\|x_{i}-{\hat{x}}_{i}\right\|}_{2}^{2}\;,$$

where $x_{i}$ is the original patch and ${\hat{x}}_{i}$ is the reconstructed patch at index i.

To facilitate a gradual increase in task difficulty, we divide the 800 pretraining epochs into four stages of 200 epochs each. In each stage, the masking ratio $r\in \left\{60\%,\;70\%,\;80\%,\;90\%\right\}$ is increased, which makes the reconstruction problem harder as the model progresses. The intuition is that earlier stages with lower masking ratios provide more contextual information, allowing the encoder to learn low-level structures and spatial priors. As the masking ratio increases, the model is forced to infer missing content from sparse cues, promoting higher-level semantic learning.

To generate multiple useful model snapshots within a single pretraining run, we employ a cyclic cosine learning rate schedule during each stage [33]. The learning rate at iteration t is defined as:

(2) $$\eta \left(t\right)=\eta_{min}+\frac{1}{2}\left(\eta_{max}-\eta_{min}\right)\left[1+cos\left(\frac{\pi t}{T}\right)\right],\;$$

where T is the number of iterations per cycle. This oscillatory schedule drives the optimizer to escape local minima and converge to distinct optima, resulting in diverse pretrained models at each cycle. These snapshots are later used in downstream ensembling strategies such as CurriMAE-Soup and CurriMAE-GE2.

To implement the pretraining procedure, we employed the AdamW optimizer with parameters $\beta_{1}=0.9,\;\;\beta_{2}=0.99$, and a weight decay of 0.05. To ensure that critical anatomical features are preserved while encouraging generalization, we applied weak image augmentations including resizing to 256×256, cropping to 224×224, and horizontal flipping. All images were normalized using the mean and standard deviation from the ImageNet dataset. The model was pretrained for 800 epochs with a batch size of 256.

### 3.3. Fine-Tuning: Downstream Classification with Three Ensemble Strategies

**Figure 3:**
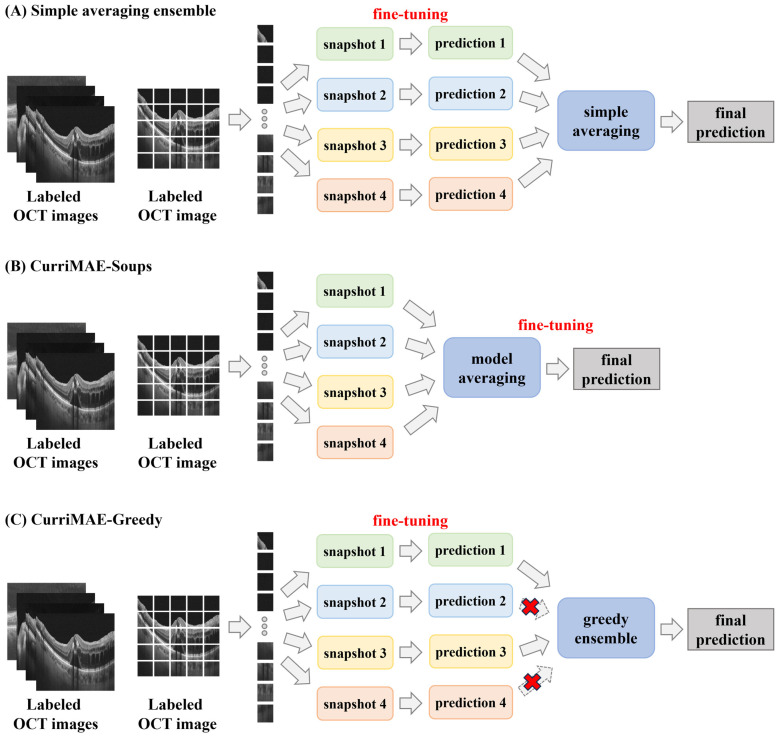
Fine-tuning and ensemble strategies. (**A**) Simple averaging: each snapshot is fine-tuned independently, and their predictions are averaged. (**B**) CurriMAE-Soup: snapshot encoders are averaged in parameter space to form a single model, which is fine-tuned once. (**C**) CurriMAE-Greedy (CurriMAE-GE2): each snapshot is fine-tuned independently, the top-2 performing models on validation are selected, and their predictions are averaged. Dashed arrows with red crosses indicate predictions that are excluded during the greedy selection process.

After pretraining the encoder using our curriculum-based progressive masking strategy, we extract model snapshots at the end of each stage. Specifically, at each of the four masking stages $r\in \left\{60\%,\;70\%,\;80\%,\;90\%\right\}$, the encoder weights $\theta_{i}\in R^{d}$ are saved, resulting in a snapshot set $\Theta =\left\{\theta_{1},\theta_{2},\theta_{3},\theta_{4}\right\}$. These snapshots encode representations learned under different reconstruction difficulties, and thus capture complementary features. To leverage these diverse models for downstream supervised classification, we apply three fine-tuning strategies, each designed to balance ensemble performance and computational efficiency. These methods are illustrated in Figure 3.

#### 3.3.1. Simple Averaging Ensemble—CurriMAE

In the CurriMAE strategy (Figure 3A), each of the four snapshot encoders is fine-tuned independently on the labeled OCTDL training set without masking. A linear classification head $f_{cls}\in R^{768\times 7}$ is attached to the [CLS] token of each model, and the classification output is computed as:

(3) $$\hat{y}=softmax\left(f_{cls}\left(z_{cls}\right)\right)\;,$$

where $z_{cls}\in R^{768}$ is the [CLS] token representation. The model is trained to minimize the standard cross-entropy loss:

(4) $$L_{CE}=-\sum_{c=1}^{C}y_{c}log\left({\hat{y}}_{c}\right)\;,$$

where $y_{c}\in \left\{0,\;1\right\}$ is the ground-truth label for class c, and ${\hat{y}}_{c}\in \left[0,\;1\right]$ is the predicted probability for class c obtained from the softmax output. C denotes the total number of classes.

During inference, predictions from all four fine-tuned models are averaged to produce the final class probability. This method maintains snapshot diversity and typically improves generalization performance, but it requires multiple fine-tuning runs and larger inference cost, as four models must be stored and evaluated.

#### 3.3.2. Parameter Averaging—CurriMAE-Soup

To reduce the cost associated with multiple fine-tuning runs, we adopt a parameter space ensembling strategy termed CurriMAE-Soup (Figure 3B). Before fine-tuning, the snapshot encoders are merged by computing their uniform layer-wise average:

(5) $$\theta_{soup}=\frac{1}{K}\sum_{i=1}^{K}\theta_{i}\;,$$

where K is the number of snapshots. In our experiments, we set K=4. The resulting soup encoder $\theta_{soup}$ is then fine-tuned only once, producing a single model used at deployment. The decoder is discarded at this stage since only the encoder is transferred for fine-tuning. This approach significantly reduces fine-tuning time, storage, and inference latency while still capturing the shared representational richness of the diverse snapshots.

#### 3.3.3. Greedy Snapshot Selection—CurriMAE-Greedy

In the CurriMAE-Greedy strategy (Figure 3C), we first fine-tuned each snapshot model independently as in CurriMAE. Their individual validation performances are then assessed, and the top- k models (based on validation AUPRC) are selected for inference. For example, in our CurriMAE-GE2 and CurriMAE-GE3 configurations, k=2 and k=3, respectively. The final prediction is obtained by averaging the outputs from these top-performing models. Although CurriMAE-Greedy imposes greater fine-tuning and inference cost compared to CurriMAE-Soup, it remains more efficient than full snapshot ensembling. By selectively excluding underperforming models, it reduces the inference burden while maintaining strong predictive performance. Notably, CurriMAE-GE2 achieves this with only two models at inference, making it attractive for resource-constrained deployment scenarios.

For all strategies, fine-tuning is conducted using the AdamW optimizer ($\beta_{1}=0.9,\;\beta_{2}=0.99$, weight decay = 0.05), with an initial learning rate of $2.5\times 10^{-3}$ decayed via cosine scheduling and a 5-epoch warm-up. We employ a layer-wise learning rate decay of 0.55, RandAugment with magnitude 6, and a DropPath rate of 0.2 [34]. Training is performed for 75 epochs, and each experiment is repeated three times with different random seeds to ensure reproducibility and robustness.

### 3.4. Computational Cost and Resource Usage

To quantify the computational requirements of each approach, we measured pretraining, fine-tuning, and inference costs in terms of number of runs, floating point operations (FLOPs) per sample, parameter counts, model size, maximum GPU memory usage, and training time per epoch as shown in Table 2. Here, m refers to the number of independently pretrained models, and k refers to the number of snapshot checkpoints generated within a single progressive pretraining run. In our experiments, we set m=4, representing separately pretrained MAE models using masking ratios of 60%, 70%, 80%, and 90%. For CurriMAE-based methods, k=4 denotes snapshots obtained from the cyclic cosine learning rate schedule at 200, 400, 600, and 800 epochs during a single pretraining run.

During pretraining, standard MAE requires m independent runs, each with a fixed masking ratio. In contrast, CurriMAE, CurriMAE-Soup, and CurriMAE-Greedy complete pretraining in a single run, varying the masking ratio progressively according to a curriculum schedule. As a result, the storage and computational cost for MAE scale with m, whereas CurriMAE methods are significantly more efficient. Table 2 emphasizes that pretraining is the most computationally demanding stage across all methods, with resource consumption far exceeding that of fine-tuning.

In the fine-tuning phase, CurriMAE-Soup is the most computationally efficient, requiring only one fine-tuning run on the model soup derived from the k snapshots. In contrast, MAE requires m fine-tuning runs, and CurriMAE and CurriMAE-Greedy require k fine-tuning runs, each initialized from a different snapshot.

For inference, CurriMAE-Soup requires only one model call, while MAE and CurriMAE require m and k models respectively. Notably, CurriMAE-Greedy reduces inference cost by selecting only two or three models from the k candidates—denoted as CurriMAE-GE2 and CurriMAE-GE3, respectively—while still leveraging the diversity of pretrained representations. This makes CurriMAE-Greedy particularly attractive for deployment in resource-constrained environments, offering ensemble-level performance with reduced inference overhead.

All experiments were conducted on a Linux workstation running Ubuntu 24.04, equipped with an Intel Core Ultra 7 265KF CPU, 128 GB RAM, and an NVIDIA RTX 5090 GPU with 32 GB of VRAM. The implementation was based on PyTorch 2.7.0 with CUDA 12.8 acceleration, and timm 0.3.2 was used for model components.

## 4. Results

### 4.1. Comparison of Supervised and Self-Supervised Baselines with CurriMAE

We report the quantitative evaluation results in Table 3, comparing supervised baselines (ResNet-34 and ViT-S), MAE with different masking ratios, and our proposed CurriMAE-based models [9,12,35,36]. The metrics used in this study include AUC, AUPRC, accuracy (ACC), sensitivity (SEN), precision (PRE), and F1-score (F1), all measured on the OCTDL test set. To account for class imbalance in this multiclass classification task, we report all metrics using weighted averaging, ensuring that the performance reflects class distribution fairly. In addition, class-wise performance results for all models are provided in Supplementary Tables S1–S4, reporting AUC, AUPRC, sensitivity, precision, and F1-score for each retinal disease category. Macro-averaged performance results, which assign equal importance to each class, are provided in Supplementary Table S5 to facilitate the assessment of minority-class reliability. All supervised and self-supervised models are built upon architectures with approximately 22 million parameters, enabling a fair comparison in terms of model capacity.

Among the supervised baselines, ViT-S trained from scratch (FS) exhibited the lowest performance across all metrics (AUC 0.907, AUPRC 0.764, F1-score 0.637), reflecting the difficulty of training ViT models without large-scale supervision. ResNet-34 (FS) showed moderately better results (AUC 0.980, AUPRC 0.906, F1-score 0.856). When pretrained on ImageNet (IN), both models improved markedly: ViT-S (IN) achieved an AUC of 0.992, AUPRC of 0.953, and F1-score of 0.911, while ResNet-34 (IN) reached comparable results with an AUC of 0.991, AUPRC of 0.950, and F1-score of 0.912. These results confirm that ImageNet-based pretraining substantially enhances supervised model performance, although they still fall short of the proposed self-supervised CurriMAE variants.

When using domain-specific pretraining on the Kermany OCT dataset (OCT), ResNet-34 (OCT) achieved an AUC of 0.989, AUPRC of 0.937, and F1-score of 0.879. In contrast, ViT-S (OCT) showed markedly lower performance, with an AUC of 0.879, AUPRC of 0.718, and F1-score of 0.606. Interestingly, both ResNet-34 (OCT) and ViT-S (OCT) underperformed compared to its ImageNet counterparts, suggesting that naïve domain pretraining may not be sufficient without appropriate architectural designs or learning strategies.

MAE models pretrained on the OCT dataset with various fixed masking ratios showed competitive and consistent performance. The best among these was MAE (60%), which achieved an AUC of 0.994, AUPRC of 0.955, and F1-score of 0.929. However, selecting the optimal masking ratio requires multiple pretraining runs, which may be computationally inefficient.

Unlike standard MAE, CurriMAE models achieve comparable or better performance with just a single pretraining run under a progressive masking strategy. CurriMAE (OCT) attained an AUC of 0.994, AUPRC of 0.956, and F1-score of 0.928, closely matching the best MAE (60%). CurriMAE-Soup reduced inference and fine-tuning cost while maintaining robust performance. Among the proposed methods, CurriMAE-GE2 and CurriMAE-GE3 achieved the highest AUC and F1-score on the OCTDL test set. While both variants showed comparable peak performance, CurriMAE-GE2 provided the best trade-off between performance and inference efficiency by requiring only two models at test time, whereas CurriMAE-GE3 required an additional model with marginal performance gains. These results indicate that the CurriMAE framework, particularly the greedy ensemble variants, provides a favorable balance between performance and efficiency compared to standard MAE models and supervised baselines.

In terms of class-wise reliability, Supplementary Tables S1–S4 show that minority classes such as RAO, RVO, and VID exhibit lower AUPRC and F1-scores with higher variability across models, reflecting the limited number of samples available for these categories. This trend is further captured by the macro-averaged results in Supplementary Table S5, where performance differences between methods become more pronounced compared to weighted averages. Notably, CurriMAE-based models demonstrate more stable macro-averaged performance, suggesting improved robustness under sever class imbalance, although reliable prediction for rare disease categories remains inherently challenging.

**Figure 4:**
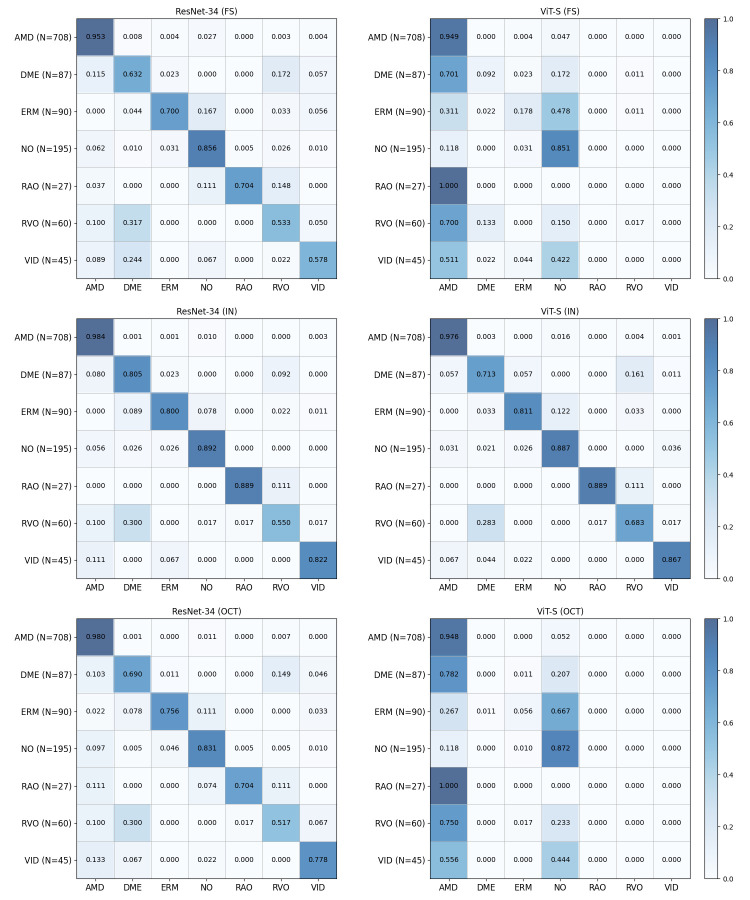
Confusion matrices of supervised baseline models (ResNet-34 and ViT-S) trained from scratch (FS), pretrained on ImageNet (IN), and pretrained on the Kermany OCT dataset (OCT) on the OCTDL test set. All confusion matrices are row-normalized (each row corresponds to the ground-truth class and each column to the predicted class) and are constructed by aggregating predictions across three random seeds; the number of test samples for each class is indicated in parentheses.

**Figure 5:**
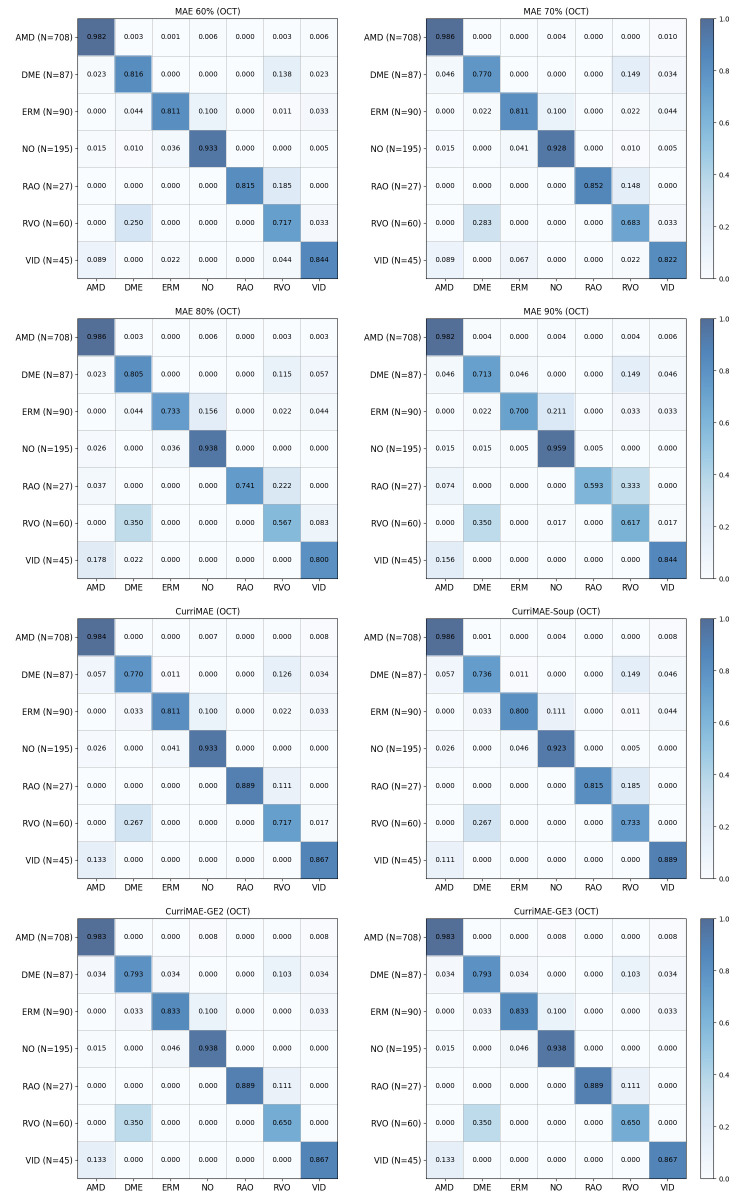
Confusion matrices of MAE variants and CurriMAE-based models on the OCTDL test set. All confusion matrices are row-normalized (each row corresponds to the ground-truth class and each column to the predicted class) and are constructed by aggregating predictions across three random seeds; the number of test samples for each class is indicated in parentheses.

To further analyze class-level prediction behavior, confusion matrices for supervised baselines and self-supervised models are presented in Figure 4 and Figure 5, respectively. All confusion matrices are row-normalized (each row corresponds to the ground-truth class and each column to the predicted class) and are constructed by aggregating predictions across three random seeds, with the number of test samples for each class indicated in parentheses. The results show that misclassifications frequently occur between clinically related categories, particularly for minority classes such as RAO, RVO, and VID, which are often confused with more prevalent retinal disease classes. Consistent with the class-wise and macro-averaged metrics, CurriMAE ensemble variants, particularly GE2 and GE3, exhibit reduced confusion patterns for minority classes compared to supervised baselines and standard MAE variants, indicating that snapshot aggregation across curriculum stages contributes to more stable class-level predictions under severe class imbalance.

### 4.2. Fixed vs. Adaptive Epoch Scheduling in CurriMAE

To investigate the impact of curriculum stage scheduling, we compared two masking strategies: a fixed-epoch schedule, where the masking ratio is increased every 200 epochs (i.e., at 200, 400, 600, 800), and an adaptive-epoch schedule, where more training epochs are allocated to stages with higher masking ratios (i.e., 60% for 125 epochs, 70% for 175, 70% for 225, and 90% for 275). This adaptive strategy devotes less time to easier reconstruction tasks and progressively increases training for harder stages to better capture complex visual patterns.

As shown in Table 4, the fixed-epoch schedule generally achieved slightly better or comparable performance across most metrics. For example, CurriMAE-GE2 trained with fixed epochs achieved an AUC of 0.995, AUPRC of 0.960, and F1-score of 0.933, while the same variant with adaptive epochs reached the same AUC but a slightly lower AUPRC of 0.956 and F1-score of 0.932. Similar trends are observed across CurriMAE and CurriMAE-Soup models. While adaptive scheduling offers a more nuanced training approach by allocating fewer resources to easier stages, our results indicate that the uniform training duration of fixed masking stages remains more effective for final classification performance. Therefore, although adaptive scheduling may provide computational benefits, fixed-stage training is preferred when performance is the primary objective.

### 4.3. Performance Comparison: CurriMAE Ensembles vs. Individually Fine-Tuned Snapshots

To further evaluate the effectiveness of our proposed CurriMAE framework, we compared its ensemble variants (CurriMAE, CurriMAE-Soup, CurriMAE-GE2, CurriMAE-GE3) against individually fine-tuned snapshot models obtained at four pretraining stages (200, 400, 600, and 800 epochs). The experiment was designed to assess whether simply fine-tuning intermediate checkpoints from the MAE pretraining process could match or surpass the performance of our ensemble-based methods.

**Figure 6:**
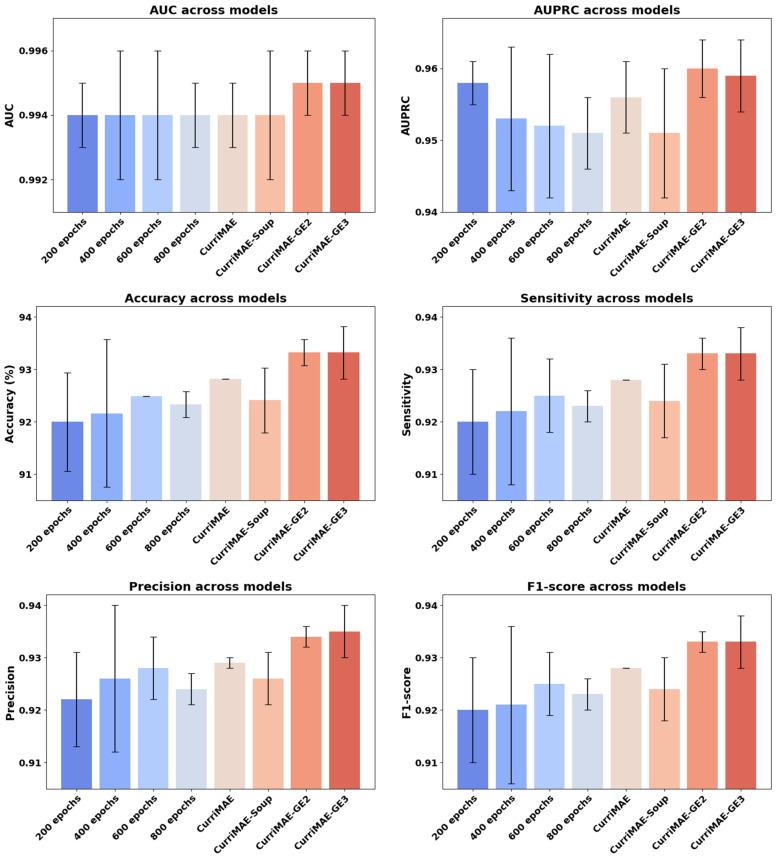
Comparison of individually fine-tuned snapshots and CurriMAE variants on OCTDL test set. Performance comparison between individually fine-tuned snapshot models (at 200, 400, 600, and 800 epochs) and CurriMAE variants (CurriMAE, CurriMAE-Soup, CurriMAE-GE2, CurriMAE-GE3) on the OCTDL test.

As shown in Figure 6, CurriMAE-GE2 and GE3 consistently achieved top-tier performance across all evaluation metrics, with the highest AUC and F1-score among the compared methods. Notably, even the strongest single snapshot model (trained at 600 epochs) fell slightly short compared to the ensemble results. These findings suggest that snapshot-based ensembles derived from curriculum-pretrained models can better leverage representations from different training stages than single snapshot models. The corresponding numerical results for these comparisons are provided in Supplementary Table S6.

### 4.4. Ablation Study

While the preceding sections report overall performance comparisons, this ablation study focuses on isolating the contribution of each component in the proposed CurriMAE pipeline using the same experimental settings.

#### 4.4.1. Effect of Curriculum-Based Progressive Masking

We first examine the impact of curriculum-based progressive masking by comparing CurriMAE with standard MAE models pretrained using fixed masking ratios. As shown in Table 3, CurriMAE achieves performance comparable to or slightly better than the best fixed-mask MAE variant while requiring only a single pretraining run. This result demonstrates that progressive masking effectively replaces the need for manual selection of an optimal masking ratio.

#### 4.4.2. Effect of Snapshot-Based Ensemble Strategies

Next, we analyze the contribution of snapshot-based ensemble strategies by comparing individually fine-tuned snapshot models with CurriMAE ensemble variants. Figure 6 shows that ensemble methods consistently outperform single snapshot models across all evaluation metrics, indicating that aggregating representations from different curriculum stages leads to more robust predictions. These results confirm the effectiveness of snapshot ensembling in the CurriMAE framework.

#### 4.4.3. Effect of Greedy Model Selection and Ensemble Size

We further investigate the effect of greedy model selection by comparing CurriMAE-GE2 and CurriMAE-GE3, which differ only in the number of ensemble members. As reported in Table 3, both variants achieve comparable performance across metrics, while Table 2 shows that GE2 requires fewer models during inference. This indicates that greedy selection with two models provides a more favorable trade-off between accuracy and computational efficiency.

#### 4.4.4. Effect of Epoch Scheduling Strategy

Finally, we evaluate the impact of epoch scheduling by comparing fixed and adaptive masking-stage schedules in CurriMAE variants. As summarized in Table 4, fixed-epoch scheduling yields slightly higher or comparable performance across most metrics compared to adaptive scheduling. This suggests that uniform training durations across curriculum stages are more effective for optimizing final classification performance.

## 5. Discussion

The growing availability of retinal OCT datasets has driven demand for scalable and efficient learning algorithms capable of extracting clinically meaningful features from high-resolution imaging data. In this study, we evaluated our proposed framework—CurriMAE-Soup and CurriMAE-Greedy—on a large-scale multiclass OCT classification task and demonstrated its superiority in both performance and efficiency compared to widely adopted architectures such as ResNet-34, ViT-S, and even the standard MAE. Notably, while ViT-S and ResNet-34 are well-established in supervised settings, they require substantial annotated data and exhibit lower generalization under limited-label regimes. Our methods, by contrast, rely on self-supervised pretraining and achieve competitive or superior downstream results with significantly reduced annotation requirements.

CurriMAE, originally introduced as a curriculum-based masked autoencoding framework, proposed a progressive masking strategy that enabled effective representation learning through a single pretraining run. While CurriMAE significantly reduced pretraining costs compared to conventional MAE pipelines, where multiple independent pretraining runs at fixed masking ratios were necessary, it still relied on multiple fine-tuned models for downstream tasks. This led to non-trivial computational overhead during both fine-tuning and inference, particularly in resource-constrained settings.

CurriMAE-Soup addresses this limitation by leveraging model soup techniques. CurriMAE-Soup fuses multiple pretrained snapshots—obtained from different stages of curriculum learning—by averaging their weights to create a single unified model. This soup-averaged is then fine-tuned once on the target dataset and deployed for inference, requiring only one model call at test time. As such, CurriMAE-Soup offers substantial gains in computational efficiency while preserving strong performance.

In contrast, CurriMAE-Greedy selectively ensembles a small subset of top-performing fine-tuned models based on validation performance. While this approach increases inference cost relative to CurriMAE-Soup, it achieves the best overall results in all evaluated metrics as demonstrated in our OCT classification experiments. Notably, CurriMAE-GE2 demonstrates strong performance while requiring only two snapshot models during inference, offering a compelling trade-off between predictive accuracy and computational burden.

The computational advantage of the CurriMAE family is clearly evidenced in Table 2. While standard MAE requires multiple independent pretraining runs to explore masking hyperparameters, all CurriMAE variants achieve competitive or superior performance with only a single pretraining schedule. CurriMAE-Soup reduces inference and fine-tuning costs by consolidating models before deployment, and CurriMAE-Greedy lowers computational load relative to full ensembling by limiting the number of inference-time models. Overall, our methods outperform standard MAE baselines in both accuracy and resource efficiency, making them more suitable for deployment in real-world clinical settings where computational budgets are constrained.

Compared to existing methods such as Snapshot Ensembles and Model Soup, which were originally designed for supervised learning, our approach is uniquely tailored for self-supervised representation learning [13,30]. Snapshot Ensembles aggregate model checkpoints across a single training cycle and have shown performance improvement through ensembling, but incur inference-time overhead due to the need for multiple model evaluations. Model Soup, on the other hand, performs weight averaging across multiple independently fine-tuned models, thereby reducing inference-time cost compared to standard ensembles. However, it typically requires multiple separate fine-tuning runs and assumes the availability of several high-performing models, which can be computationally demanding in self-supervised settings. By contrast, our approach begins with a single progressive pretraining run, producing multiple curriculum-aware checkpoints. These checkpoints are then either fused into a single efficient model (CurriMAE-Soup) or selectively combined through a greedy ensemble strategy (CurriMAE-Greedy), offering distinct advantages in terms of both deployment efficiency and predictive performance. Importantly, this framework retains the scalability and robustness of ensemble methods while mitigating computational burden, making it particularly well-suited for medical imaging applications where inference efficiency and limited computational resources are often critical concerns.

Despite the notable advantages, several limitations remain. In CurriMAE-Soup, the fused model is constructed by averaging checkpoints that originated from different pretraining stages rather than from the same initialization, unlike classical Model Soup approaches. As a result, the averaged weights may not reside near an optimal local minimum, which can affect fine-tuning stability. CurriMAE-Greedy, while delivering strong performance, still requires multiple model calls at inference, which may limit its use in latency-sensitive environments. These trade-offs emphasize that CurriMAE-Soup is better suited for highly constrained deployments, whereas CurriMAE-Greedy may be preferable in applications where inference performance is paramount. These limitations point out that while both CurriMAE-Soup and CurriMAE-Greedy provide meaningful improvements in either computational efficiency or predictive performance, they do not simultaneously achieve optimality on both fronts. Future work could explore hybrid approaches that better align the pretraining trajectories of snapshots used for fusion or ensembling, and develop lightweight adapters to bridge the discrepancies between diverse pretrained models without requiring full retraining.

## 6. Conclusions

This study presents a novel ensemble framework that integrates curriculum-based SSL with efficient model fusion and selection strategies for retinal OCT classification. CurriMAE-Soup and CurriMAE-Greedy provide flexible pathways to optimize for either computational efficiency or predictive performance without requiring multiple independent pretraining runs. Our methods outperform both standard MAE and widely used supervised baselines in accuracy and resource consumption, demonstrating strong potential for real-world ophthalmic imaging applications.

## Figures and Tables

**Table 1:** Summary of datasets used for pretraining and fine-tuning phases.

Phases	Dataset Name	Classe Labels ^†^	Number of Pretraining Data	Number of Train Data	Number of Validation Data *	Number of Test Data
Pretraining	Kermany	-	108,309	-	-	-
Fine-tuning	OCTDL (7 classes, Total = 2064)	AMD	-	796	199	236
		NOR	-	214	53	65
		ERM	-	100	25	30
		DME	-	94	24	29
		RVO	-	65	16	20
		VID	-	49	12	15
		RAO	-	10	3	9

* For the OCTDL dataset, 20% of the training set was randomly held out for validation. ^†^ Class labels denote retinal disease categories: AMD (Age-related Macular Degeneration), NOR (Normal), ERM (Epiretinal Membrane), DME (Diabetic Macular Edema), RVO (Retinal Vein Occlusion), VID (Vitreomacular Interface Disease), and RAO (Retinal Artery Occlusion).

**Table 2:** Computational cost and resource usage for pretraining, fine-tuning, and inference across MAE, CurriMAE, CurriMAE-Soup, and CurriMAE-GE2.

Phase	Metric	MAE	CurriMAE	CurriMAE-Soup	CurriMAE-GE2
Pretraining	Pretraining runs	m *	1	1	1
	FLOPs per sample (G)	1.78	1.78	1.78	1.78
	Parameters (Millions)	22.14	22.14	22.14	22.14
	Model Size (MB)	84.89 × m	84.89	84.89	84.89
	Max GPU Memory Usage (MB)	1639.89	1639.89	1639.89	1639.89
	Training Time per Epoch (min:sec)	4:36 × m	4:30	4:30	4:30
Fine-tuning	Fine-tuning runs	m	k ^+^	1	k
	FLOPs per sample (G)	6.44	6.44	6.44	6.44
	Parameters (Millions)	21.67	21.67 × k	21.67	21.67 × k
	Model Size (MB)	82.71 × m	82.71 × k	82.71	82.71 × k
	Max GPU Memory Usage (MB)	577.78	577.78	577.78	577.78
	Total Training Time (min:sec)	1:47 × m	1:49 × k	1:48	1:48 × k
Inference	Inference models required	m	k	1	2
	Inference FLOPs per sample (G)	6.44 × m	6.44 × k	6.44	6.44 × 2

* m refers to the number of pretrained MAE models generated with different masking ratios. ^+^ k denotes the number of fine-tuned models derived from curriculum-based snapshots (e.g., at 200, 400, 600, and 800 epochs).

**Table 3:** Performance comparison of supervised baselines, MAE variants, and CurriMAE methods on the OCTDL test set. Each model is evaluated using AUC, AUPRC, accuracy (ACC), sensitivity (SEN), precision (PRE), and F1-score. All values are reported as mean (standard deviation) across three runs. Best results in each metric are shown in bold.

Models	AUC	AUPRC	ACC	SEN	PRE	F1
ResNet-34 (FS) *	0.980 (0.002)	0.906 (0.009)	85.56 (0.62)	0.855 (0.006)	0.860 (0.006)	0.856 (0.005)
ViT-S (FS)	0.907 (0.013)	0.764 (0.014)	71.20 (1.36)	0.712 (0.014)	0.625 (0.036)	0.637 (0.028)
ResNet-34 (IN) ^+^	0.991 (0.003)	0.950 (0.012)	91.34 (2.62)	0.913 (0.026)	0.914 (0.026)	0.912 (0.027)
ViT-S (IN)	0.992 (0.001)	0.953 (0.006)	91.01 (1.00)	0.910 (0.010)	0.914 (0.008)	0.911 (0.009)
ResNet-34 (OCT) **	0.989 (0.001)	0.937 (0.004)	88.20 (0.14)	0.882 (0.002)	0.881 (0.004)	0.879 (0.003)
ViT-S (OCT)	0.879 (0.021)	0.718 (0.024)	69.80 (1.28)	0.698 (0.013)	0.544 (0.030)	0.606 (0.017)
MAE 60% (OCT)	0.994 (0.001)	0.955 (0.005)	92.74 (1.03)	0.928 (0.010)	0.931 (0.010)	0.929 (0.010)
MAE 70% (OCT)	0.993 (0.002)	0.953 (0.006)	92.41 (0.94)	0.924 (0.010)	0.927 (0.007)	0.925 (0.009)
MAE 80% (OCT)	0.994 (0.001)	0.943 (0.006)	91.34 (0.66)	0.913 (0.007)	0.917 (0.006)	0.912 (0.010)
MAE 90% (OCT)	0.991 (0.002)	0.938 (0.010)	91.75 (1.74)	0.906 (0.011)	0.911 (0.012)	0.916 (0.018)
CurriMAE (OCT)	0.994 (0.001)	0.956 (0.005)	92.82 (0.00)	0.928 (0.000)	0.929 (0.001)	0.928 (0.000)
CurriMAE-Soup (OCT)	0.994 (0.002)	0.951 (0.009)	92.41 (0.62)	0.924 (0.007)	0.926 (0.005)	0.924 (0.006)
CurriMAE-GE2 (OCT)	**0.995** **(0.001)**	**0.960** **(0.004)**	**93.32** **(0.25)**	**0.933** **(0.003)**	0.934 (0.002)	**0.933** **(0.002)**
CurriMAE-GE3 (OCT)	**0.995** **(0.001)**	0.959 (0.005)	**93.32** **(0.50)**	**0.933** **(0.005)**	**0.935** **(0.005)**	**0.933** **(0.005)**

* “FS” denotes models trained from scratch. ^+^ “IN” refers to models pretrained on ImageNet. ** “OCT” refers to models pretrained on the Kermany dataset.

**Table 4:** Comparison of CurriMAE variants using fixed versus adaptive epoch schedules on the OCTDL test set. All values are reported as mean (standard deviation) across three runs. Best results in each metric are shown in bold.

	Models	AUC	AUPRC	ACC	SEN	PRE	F1
Fixed epochs	CurriMAE (OCT)	0.994 (0.001)	0.956 (0.005)	92.82 (0.00)	0.928 (0.000)	0.929 (0.001)	0.928 (0.000)
	CurriMAE-Soup (OCT)	0.994 (0.002)	0.951 (0.009)	92.41 (0.62)	0.924 (0.007)	0.926 (0.005)	0.924 (0.006)
	CurriMAE-GE2 (OCT)	**0.995** **(0.001)**	**0.960** **(0.004)**	**93.32** **(0.25)**	**0.933** **(0.003)**	0.934 (0.002)	**0.933** **(0.002)**
	CurriMAE-GE3 (OCT)	**0.995** **(0.001)**	0.959 (0.005)	**93.32** **(0.50)**	**0.933** **(0.005)**	**0.935** **(0.005)**	**0.933** **(0.005)**
Adaptive epochs	CurriMAE (OCT)	0.993 (0.001)	0.952 (0.002)	92.90 (0.14)	0.929 (0.002)	0.930 (0.001)	0.929 (0.001)
	CurriMAE-Soup (OCT)	0.993 (0.002)	0.948 (0.008)	91.58 (0.25)	0.916 (0.003)	0.919 (0.003)	0.916 (0.002)
	CurriMAE-GE2 (OCT)	**0.995** **(0.001)**	0.956 (0.001)	93.23 (0.57)	**0.933** **(0.006)**	0.933 (0.006)	0.932 (0.006)
	CurriMAE-GE3 (OCT)	**0.995** **(0.001)**	0.956 (0.002)	93.15 (0.52)	0.932 (0.005)	0.932 (0.007)	0.931 (0.006)

## Data Availability

The data presented in this study are available in Mendeley Data at https://data.mendeley.com/, reference numbers [14,32]. These data were derived from the following resources available in the public domain: Kermany dataset—https://data.mendeley.com/datasets/rscbjbr9sj/2 (accessed on 30 November 2025), OCTDL dataset—https://data.mendeley.com/datasets/sncdhf53xc/4 (accessed on 30 November 2025).

## Supplementary Materials

The following supporting information can be downloaded at https://www.mdpi.com/article/10.3390/diagnostics16020179/s1, Table S1. Class-wise AUC, AUPRC, sensitivity (SEN), precision (PRE), and F1-score (F1) of ResNet-34 (FS) and ViT-S (FS) on the OCTDL test set. Table S2. Class-wise AUC, AUPRC, sensitivity (SEN), precision (PRE), and F1-score (F1) of ResNet-34 (IN), ViT-S (IN), ResNet-34 (OCT), ViT-S (OCT) on the OCTDL test set. Table S3. Class-wise AUC, AUPRC, sensitivity (SEN), precision (PRE), and F1-score (F1) of MAE 60%, MAE 70%, MAE 80%, and MAE 90% on the OCTDL test set. Table S4. Class-wise AUC, AUPRC, sensitivity (SEN), precision (PRE), and F1-score (F1) of CurriMAE, CurriMAE-soup, CurriMAE-GE2, CurriMAE-GE3 on the OCTDL test set. Table S5. Macro-averaged performance comparison of supervised baselines, MAE variants, and CurriMAE methods on the OCTDL test set. Each model is evaluated using AUC, AUPRC, accuracy (ACC), sensitivity (SEN), precision (PRE), and F1-score. All values are reported as mean (standard deviation) across three runs. Best results in each metric are shown in bold. Table S6. Comparison of snapshot-based fine-tuning and CurriMAE variants on OCTDL test set. Best results in each metric are shown in bold.

## Abbreviations

The following abbreviations are used in this manuscript:

ACC	Accuracy
AUC	Area under the receiver operating characteristic curve
AUPRC	Area under the precision–recall curve
BERT	Bidirectional encoder representations from transformers
CNN	Convolutional neural networks
CNV	Choroidal neovascularization
CurriMAE	Curriculum learning-based masked autoencoders
DME	Diabetic macular edema
ERM	Epiretinal membrane
FLOPs	Floating point operations
F1	F1-score
MAE	Masked autoencoders
MIM	Masked image modeling
MSE	Mean squared error
NOR	Normal
OCT	Optical coherence tomography
PRE	Precision
RAO	Retinal artery occlusion
RVO	Retinal vein occlusion
SEN	Sensitivity
Snap-MAE	Snapshot ensemble learning-based masked autoencoders
SSL	Self-supervised learning
VID	Vitreomacular interface disease
ViT	Vision transformer
