# Supplementary material for: Efficient Ensemble Learning with Curriculum-Based Masked Autoencoders for Retinal OCT Classification
Source: Diagnostics (Basel). 2026 Jan 6;16(2):179. doi: 10.3390/diagnostics16020179 (PMC12840412; doi:10.3390/diagnostics16020179)
Supplement: Supplementary file 1 [file diagnostics-16-00179-s001.zip › diagnostics-4047868-supplementary.pdf]

Efficient Ensemble Learning with Curriculum-Based Masked Autoencoders for Retinal OCT Classification

Taeyoung Yoon, Daesung Kang*

School of Bio-Health Convergence, College of Natural Sciences, Sungshin Women's University, Seoul, Republic of Korea

Table S1. Class-wise AUC, AUPRC, sensitivity (SEN), precision (PRE), and F1-score (F1) of **ResNet-34 (FS) and ViT-S (FS)** on the OCTDL test set.

	Classes†	AUC	AUPRC	SEN	PRE	F1
ResNet-34 (OCT)	AMD	0.988 (0.001)	0.992 (0.001)	0.953 (0.013)	0.953 (0.010)	0.953 (0.005)
	DME	0.955 (0.004)	0.631 (0.028)	0.632 (0.040)	0.567 (0.016)	0.598 (0.027)
	ERM	0.984 (0.002)	0.864 (0.013)	0.700 (0.033)	0.854 (0.052)	0.768 (0.026)
	NOR	0.979 (0.002)	0.903 (0.011)	0.857 (0.023)	0.807 (0.019)	0.831 (0.018)
	RAO	0.972 (0.014)	0.826 (0.033)	0.704 (0.064)	0.952 (0.083)	0.808 (0.063)
	RVO	0.957 (0.014)	0.612 (0.076)	0.533 (0.076)	0.522 (0.056)	0.524 (0.041)
	VID	0.936 (0.026)	0.635 (0.122)	0.578 (0.102)	0.590 (0.009)	0.581 (0.055)
ViT-S (FS)	AMD	0.922 (0.015)	0.946 (0.01)	0.949 (0.004)	0.768 (0.029)	0.849 (0.017)
	DME	0.835 (0.018)	0.350 (0.057)	0.092 (0.130)	0.471 (0.503)	0.124 (0.160)
	ERM	0.884 (0.015)	0.465 (0.051)	0.178 (0.069)	0.546 (0.050)	0.264 (0.085)
	NOR	0.943 (0.010)	0.738 (0.031)	0.851 (0.050)	0.582 (0.017)	0.692 (0.027)
	RAO	0.903 (0.039)	0.533 (0.023)	0.000 (0.000)	0.000 (0.000)	0.000 (0.000)
	RVO	0.853 (0.038)	0.355 (0.073)	0.017 (0.029)	0.167 (0.289)	0.030 (0.053)
	VID	0.769 (0.023)	0.107 (0.013)	0.000 (0.000)	0.000 (0.000)	0.000 (0.000)

† Classes denote retinal disease categories: AMD (Age-related Macular Degeneration), DME (Diabetic Macular Edema), ERM (Epiretinal Membrane), NOR (Normal), RAO (Retinal Artery Occlusion), RVO (Retinal Vein Occlusion), and VID (Vitreomacular Interface Disease).

Table S2. Class-wise AUC, AUPRC, sensitivity (SEN), precision (PRE), and F1-score (F1) of **ResNet-34 (IN), ViT-S (IN), ResNet-34 (OCT), ViT-S (OCT)** on the OCTDL test set.

	**Classes**	**AUC**	**AUPRC**	**SEN**	**PRE**	**F1**
ResNet-34 (IN)	AMD	0.997 (0.002)	0.998 (0.002)	0.985 (0.009)	0.960 (0.029)	0.972 (0.019)
	DME	0.983 (0.005)	0.777 (0.057)	0.805 (0.040)	0.690 (0.075)	0.742 (0.056)
	ERM	0.985 (0.007)	0.908 (0.035)	0.800 (0.088)	0.872 (0.058)	0.831 (0.046)
	NOR	0.993 (0.003)	0.967 (0.008)	0.892 (0.093)	0.921 (0.009)	0.904 (0.048)
	RAO	0.946 (0.035)	0.895 (0.004)	0.889 (0.000)	0.963 (0.064)	0.924 (0.030)
	RVO	0.960 (0.012)	0.711 (0.057)	0.550 (0.100)	0.720 (0.031)	0.619 (0.061)
	VID	0.989 (0.004)	0.899 (0.020)	0.822 (0.039)	0.910 (0.101)	0.862 (0.054)
ViT-S (IN)	AMD	0.998 (0.001)	0.998 (0.001)	0.976 (0.005)	0.980 (0.006)	0.978 (0.001)
	DME	0.981 (0.007)	0.809 (0.042)	0.713 (0.040)	0.690 (0.039)	0.701 (0.033)
	ERM	0.986 (0.003)	0.903 (0.013)	0.811 (0.019)	0.869 (0.021)	0.839 (0.020)
	NOR	0.992 (0.001)	0.961 (0.004)	0.887 (0.062)	0.887 (0.009)	0.886 (0.027)
	RAO	0.944 (0.009)	0.894 (0.001)	0.889 (0.000)	0.963 (0.064)	0.924 (0.030)
	RVO	0.973 (0.008)	0.718 (0.037)	0.683 (0.058)	0.654 (0.099)	0.663 (0.036)
	VID	0.994 (0.001)	0.947 (0.010)	0.866 (0.115)	0.837 (0.185)	0.835 (0.074)
ResNet-34 (OCT)	AMD	0.993 (0.001)	0.996 (0.001)	0.981 (0.010)	0.939 (0.006)	0.959 (0.002)
	DME	0.975 (0.003)	0.752 (0.021)	0.690 (0.120)	0.671 (0.037)	0.675 (0.054)
	ERM	0.990 (0.002)	0.910 (0.009)	0.755 (0.069)	0.873 (0.040)	0.809 (0.042)
	NOR	0.989 (0.002)	0.949 (0.011)	0.831 (0.031)	0.889 (0.055)	0.857 (0.010)
	RAO	0.967 (0.006)	0.829 (0.029)	0.704 (0.064)	0.911 (0.078)	0.791 (0.038)
	RVO	0.967 (0.003)	0.628 (0.014)	0.517 (0.029)	0.588 (0.038)	0.549 (0.008)
	VID	0.980 (0.013)	0.857 (0.058)	0.778 (0.077)	0.741 (0.119)	0.755 (0.077)
ViT-S (OCT)	AMD	0.900 (0.018)	0.928 (0.013)	0.948 (0.006)	0.760 (0.012)	0.843 (0.009)
	DME	0.788 (0.032)	0.309 (0.049)	0.000 (0.000)	0.000 (0.000)	0.000 (0.000)
	ERM	0.842 (0.025)	0.351 (0.012)	0.056 (0.096)	0.185 (0.321)	0.085 (0.148)
	NOR	0.939 (0.011)	0.692 (0.048)	0.872 (0.058)	0.534 (0.026)	0.661 (0.030)
	RAO	0.712 (0.085)	0.076 (0.062)	0.000 (0.000)	0.000 (0.000)	0.000 (0.000)
	RVO	0.760 (0.036)	0.202 (0.119)	0.000 (0.000)	0.000 (0.000)	0.000 (0.000)
	VID	0.792 (0.050)	0.126 (0.035)	0.000 (0.000)	0.000 (0.000)	0.000 (0.000)

Table S3. Class-wise AUC, AUPRC, sensitivity (SEN), precision (PRE), and F1-score (F1) of **MAE 60%, MAE 70%, MAE 80%, and MAE 90%** on the OCTDL test set.

	**Classes**	**AUC**	**AUPRC**	**SEN**	**PRE**	**F1**
MAE 60% (OCT)	AMD	0.998 (0.001)	0.999 (0.001)	0.982 (0.014)	0.987 (0.007)	0.984 (0.005)
	DME	0.987 (0.002)	0.780 (0.050)	0.816 (0.040)	0.756 (0.013)	0.784 (0.020)
	ERM	0.982 (0.005)	0.900 (0.013)	0.811 (0.019)	0.893 (0.052)	0.849 (0.014)
	NOR	0.994 (0.001)	0.973 (0.002)	0.933 (0.009)	0.933 (0.009)	0.933 (0.009)
	RAO	0.989 (0.008)	0.916 (0.017)	0.815 (0.064)	1.000 (0.000)	0.897 (0.038)
	RVO	0.976 (0.003)	0.748 (0.028)	0.717 (0.058)	0.662 (0.048)	0.688 (0.050)
	VID	0.997 (0.001)	0.943 (0.014)	0.845 (0.039)	0.767 (0.093)	0.802 (0.056)
MAE 70% (OCT)	AMD	0.998 (0.001)	0.999 (0.001)	0.986 (0.002)	0.984 (0.002)	0.985 (0.002)
	DME	0.988 (0.004)	0.811 (0.064)	0.770 (0.020)	0.780 (0.030)	0.775 (0.014)
	ERM	0.974 (0.010)	0.884 (0.006)	0.811 (0.019)	0.870 (0.036)	0.839 (0.024)
	NOR	0.993 (0.002)	0.968 (0.004)	0.928 (0.009)	0.938 (0.000)	0.933 (0.005)
	RAO	0.993 (0.008)	0.936 (0.034)	0.852 (0.064)	1.000 (0.000)	0.919 (0.038)
	RVO	0.974 (0.009)	0.736 (0.037)	0.683 (0.104)	0.650 (0.044)	0.665 (0.064)
	VID	0.992 (0.004)	0.892 (0.031)	0.822 (0.039)	0.698 (0.116)	0.752 (0.074)
MAE 80% (OCT)	AMD	0.999 (0.001)	0.999 (0.000)	0.986 (0.007)	0.978 (0.008)	0.982 (0.001)
	DME	0.986 (0.004)	0.757 (0.060)	0.805 (0.163)	0.728 (0.075)	0.753 (0.057)
	ERM	0.981 (0.006)	0.875 (0.015)	0.733 (0.034)	0.905 (0.017)	0.809 (0.015)
	NOR	0.994 (0.002)	0.972 (0.011)	0.938 (0.016)	0.911 (0.027)	0.924 (0.012)
	RAO	0.986 (0.006)	0.904 (0.009)	0.741 (0.064)	1.000 (0.000)	0.850 (0.043)
	RVO	0.969 (0.012)	0.674 (0.080)	0.567 (0.153)	0.628 (0.011)	0.589 (0.094)
	VID	0.985 (0.011)	0.813 (0.060)	0.800 (0.000)	0.696 (0.060)	0.743 (0.034)
MAE 90% (OCT)	AMD	0.996 (0.001)	0.998 (0.001)	0.982 (0.006)	0.978 (0.006)	0.980 (0.005)
	DME	0.979 (0.004)	0.709 (0.044)	0.713 (0.053)	0.688 (0.071)	0.698 (0.034)
	ERM	0.976 (0.010)	0.881 (0.046)	0.700 (0.067)	0.926 (0.053)	0.797 (0.061)
	NOR	0.993 (0.001)	0.959 (0.003)	0.959 (0.018)	0.891 (0.025)	0.923 (0.010)
	RAO	0.989 (0.004)	0.869 (0.040)	0.593 (0.232)	0.958 (0.072)	0.708 (0.181)
	RVO	0.964 (0.006)	0.662 (0.009)	0.617 (0.058)	0.573 (0.035)	0.592 (0.013)
	VID	0.982 (0.016)	0.886 (0.065)	0.845 (0.039)	0.776 (0.141)	0.805 (0.081)

Table S4. Class-wise AUC, AUPRC, sensitivity (SEN), precision (PRE), and F1-score (F1) of **CurriMAE, CurriMAE-soup, CurriMAE-GE2, CurriMAE-GE3** on the OCTDL test set.

	Classes	AUC	AUPRC	SEN	PRE	F1
CurriMAE (OCT)	AMD	0.998 (0.001)	0.999 (0.001)	0.985 (0.007)	0.978 (0.006)	0.981 (0.002)
	DME	0.988 (0.002)	0.812 (0.032)	0.770 (0.040)	0.781 (0.035)	0.775 (0.016)
	ERM	0.982 (0.003)	0.890 (0.012)	0.811 (0.019)	0.891 (0.031)	0.849 (0.008)
	NOR	0.993 (0.000)	0.971 (0.001)	0.933 (0.023)	0.929 (0.007)	0.931 (0.009)
	RAO	0.977 (0.004)	0.900 (0.002)	0.889 (0.000)	1.000 (0.000)	0.941 (0.000)
	RVO	0.973 (0.009)	0.763 (0.043)	0.717 (0.076)	0.733 (0.035)	0.722 (0.021)
	VID	0.995 (0.001)	0.914 (0.024)	0.867 (0.000)	0.754 (0.065)	0.805 (0.037)
CurriMAE-soup (OCT)	AMD	0.997 (0.002)	0.998 (0.001)	0.986 (0.005)	0.979 (0.007)	0.982 (0.003)
	DME	0.984 (0.004)	0.745 (0.067)	0.736 (0.040)	0.768 (0.069)	0.749 (0.033)
	ERM	0.986 (0.002)	0.894 (0.011)	0.800 (0.033)	0.878 (0.023)	0.837 (0.028)
	NOR	0.994 (0.001)	0.972 (0.005)	0.923 (0.015)	0.933 (0.008)	0.928 (0.005)
	RAO	0.992 (0.006)	0.921 (0.016)	0.815 (0.064)	1.000 (0.000)	0.897 (0.038)
	RVO	0.973 (0.005)	0.749 (0.023)	0.733 (0.076)	0.687 (0.008)	0.708 (0.039)
	VID	0.995 (0.001)	0.908 (0.043)	0.889 (0.038)	0.745 (0.059)	0.809 (0.027)
CurriMAE-GE2 (OCT)	AMD	0.999 (0.001)	0.999 (0.001)	0.988 (0.008)	0.980 (0.002)	0.984 (0.004)
	DME	0.989 (0.003)	0.830 (0.044)	0.805 (0.020)	0.788 (0.036)	0.796 (0.01)
	ERM	0.982 (0.003)	0.896 (0.010)	0.811 (0.019)	0.891 (0.033)	0.849 (0.018)
	NOR	0.994 (0.002)	0.972 (0.003)	0.933 (0.023)	0.933 (0.007)	0.933 (0.01)
	RAO	0.977 (0.003)	0.900 (0.001)	0.889 (0.000)	1.000 (0.000)	0.941 (0.000)
	RVO	0.973 (0.004)	0.768 (0.008)	0.733 (0.076)	0.737 (0.036)	0.732 (0.026)
	VID	0.996 (0.001)	0.909 (0.048)	0.867 (0.000)	0.800 (0.073)	0.831 (0.040)
CurriMAE-GE3 (OCT)	AMD	0.999 (0.001)	0.999 (0.001)	0.984 (0.002)	0.982 (0.002)	0.983 (0.002)
	DME	0.988 (0.002)	0.825 (0.025)	0.793 (0.035)	0.786 (0.036)	0.789 (0.018)
	ERM	0.982 (0.004)	0.892 (0.013)	0.811 (0.019)	0.913 (0.017)	0.859 (0.003)
	NOR	0.993 (0.001)	0.972 (0.001)	0.954 (0.000)	0.934 (0.008)	0.944 (0.005)
	RAO	0.976 (0.005)	0.900 (0.002)	0.889 (0.000)	1.000 (0.000)	0.941 (0.000)
	RVO	0.973 (0.009)	0.761 (0.044)	0.717 (0.076)	0.746 (0.058)	0.728 (0.031)
	VID	0.995 (0.002)	0.915 (0.027)	0.867 (0.000)	0.738 (0.047)	0.796 (0.027)

Table S5. Macro-averaged performance comparison of supervised baselines, MAE variants, and CurriMAE methods on the OCTDL test set. Each model is evaluated using AUC, AUPRC, accuracy (ACC), sensitivity (SEN), precision (PRE), and F1-score. All values are reported as mean (standard deviation) across three runs. Best results in each metric are shown in bold.

Models	AUC	AUPRC	ACC	SEN	PRE	F1
ResNet-34 (FS)	0.967 (0.006)	0.780 (0.030)	85.56 (0.62)	0.708 (0.026)	0.749 (0.013)	0.723 (0.019)
ViT-S (FS)	0.873 (0.014)	0.499 (0.015)	71.20 (1.36)	0.298 (0.029)	0.362 (0.071)	0.280 (0.040)
ResNet-34 (IN)	0.979 (0.005)	0.879 (0.024)	91.34 (2.62)	0.820 (0.034)	0.862 (0.025)	0.836 (0.032)
ViT-S (IN)	0.981 (0.003)	0.890 (0.013)	91.01 (1.00)	0.832 (0.014)	0.840 (0.033)	0.833 (0.023)
ResNet-34 (OCT)	0.980 (0.002)	0.846 (0.013)	88.20 (0.14)	0.751 (0.023)	0.802 (0.010)	0.771 (0.011)
ViT-S (OCT)	0.819 (0.031)	0.383 (0.035)	69.80 (1.28)	0.268 (0.014)	0.211 (0.049)	0.227 (0.024)
MAE 60% (OCT)	**0.989 (0.002)**	0.894 (0.010)	92.74 (1.03)	0.846 (0.010)	0.857 (0.026)	0.849 (0.014)
MAE 70% (OCT)	0.987 (0.003)	0.889 (0.014)	92.41 (0.94)	0.836 (0.026)	0.846 (0.022)	0.838 (0.026)
MAE 80% (OCT)	0.986 (0.003)	0.856 (0.027)	91.34 (0.66)	0.796 (0.019)	0.835 (0.012)	0.807 (0.020)
MAE 90% (OCT)	0.983 (0.004)	0.852 (0.025)	90.59 (1.13)	0.772 (0.048)	0.827 (0.014)	0.786 (0.043)
CurriMAE (OCT)	0.987 (0.002)	0.892 (0.012)	92.82 (0.00)	0.853 (0.002)	0.866 (0.006)	0.858 (0.004)
CurriMAE-Soup (OCT)	**0.989 (0.002)**	0.884 (0.021)	92.41 (0.62)	0.840 (0.016)	0.856 (0.020)	0.844 (0.020)
CurriMAE-GE2 (OCT)	0.987 (0.001)	**0.896 (0.011)**	**93.32 (0.25)**	**0.861 (0.004)**	**0.876 (0.008)**	**0.867 (0.003)**
CurriMAE-GE3 (OCT)	0.987 (0.002)	0.895 (0.013)	**93.32 (0.50)**	0.859 (0.010)	0.871 (0.011)	0.863 (0.011)

Table S6. Comparison of snapshot-based fine-tuning and CurriMAE variants on OCTDL test set. Best results in each metric are shown in bold.

Models	AUC	AUPRC	ACC	SEN	PRE	F1
200 epochs	0.994 (0.001)	0.958 (0.003)	92.00 (0.94)	0.920 (0.010)	0.922 (0.009)	0.920 (0.010)
400 epochs	0.994 (0.002)	0.953 (0.010)	92.16 (1.41)	0.922 (0.014)	0.926 (0.014)	0.921 (0.015)
600 epochs	0.994 (0.002)	0.952 (0.010)	92.49 (0.00)	0.925 (0.007)	0.928 (0.006)	0.925 (0.006)
800 epochs	0.994 (0.001)	0.951 (0.005)	92.33 (0.25)	0.923 (0.003)	0.924 (0.003)	0.923 (0.003)
CurriMAE (OCT)	0.994 (0.001)	0.956 (0.005)	92.82 (0.00)	0.928 (0.000)	0.929 (0.001)	0.928 (0.000)
CurriMAE-Soup (OCT)	0.994 (0.002)	0.951 (0.009)	92.41 (0.62)	0.924 (0.007)	0.926 (0.005)	0.924 (0.006)
CurriMAE-GE2 (OCT)	**0.995 (0.001)**	**0.960 (0.004)**	**93.32 (0.25)**	**0.933 (0.003)**	0.934 (0.002)	**0.933 (0.002)**
CurriMAE-GE3 (OCT)	**0.995 (0.001)**	0.959 (0.005)	**93.32 (0.50)**	**0.933 (0.005)**	**0.935 (0.005)**	**0.933 (0.005)**
